# Education in Food Economy

**Published:** 1918-03-16

**Authors:** 


### EDUCATION IN FOOD ECONOMY.

### By A SOCIAL WORKER.

THAT the care of food should have begun three years ago is an unfortunate truism. That national education in its management should have begun long before is a truth now being pressed home at the sword's point. A system of voluntary rations has been tried, and the results have been remarkable as showing that people could maintain an excellent average of health on a considerable reduction of general consumption. Indeed, some of the figures reported by Dr. Hamer and others were distinctly encouraging, especially as regards children at boarding schools. Possibly there has been in some cases more attention to the food, so that the quantity received by the child was actually greater and the quality better, but certainly the maintenance of strength and the gains in weight sound satisfactory. The "chuck-tuck" leagues of boys will be wholesome to mind and body *if* the table meals are adequate.

#### A UNIVERSAL FOOD SHORTAGE.

Compulsory rationing for certain foods is now in force, and it is "up to" both those who have wanted it and those who hoped to defer it indefinitely to act upon it loyally. Successful application of the London scheme will greatly facilitate its extension. Meanwhile other great towns will perhaps follow the example of Birmingham and carry out their own "voluntary compulsion." In some of the remoter parts of the country the principle of "Eat while you can" is certainly having occasional scandalous results. It is to be hoped that, where it applies, compulsion will overcome the dull resistance of ignorance. It is easy for each section of the community to accuse another of indulgence and waste. Educated people point to camps and canteens, and to the wastefulness of servants and school-children; the working classes reply by pointing to the Ritz and the Carlton, and to the much worse example of provincial hotels. What the uneducated need to be helped to realise is that shortage of food is not merely local but universal. Millions of men and women have been turned from producers to destroyers, and as regards corn, the reserves of a year of good harvest have had to supply the deficiency of a lean year. Another poor harvest would—even apart from the war—have had ill-effects. Further, the fighting men everywhere have had to be raised to a higher scale of living. And, most important of all perhaps for us, not merely has tonnage been terribly reduced, but ships will be increasingly needed to convey men—notably the American Army—and food for their sole use. The figures given by Sir Arthur Yapp on December 4 to a gathering of the Food Ministry's accredited speakers were 663 ships of 1,600 tons and over, and 247 of less size. Supposing a liner to make five voyages yearly, this might mean 10,000,000 tons of corn brought over. Or it might be said that a liner could carry 4,500 tons of food or 1,200 to 1,300 men. It must also be remembered that France and Italy, now unable to produce as usual, must import more if they are to continue to carry on the war.

Rationing may be restricted to only a few articles of food if, but only if, every individual of the nation will determine not to trifle wth the situation or to wait for someone else to begin, but will at once throw his or her best energies into the task, one, after all, not much less important than that of the soldier. Indeed, it would be well if all would realise how gravely depressing to soldiers returning on brief leave from the mud and blood of the Front, the horrors of a push, or the weeks of weary waiting to advance, have been the signs of indifference in England, the display of quails in aspic in shop windows, the continuance of trade in finery and useless luxuries. Why curl ostrich feathers when starvation stares you in the face?

#### HOW TO COOK A CABBAGE.

The petty daily waste of small households in food ill-managed and ill-cooked is the worst source of shortage, and the place where it can be most effectively remedied. A real knowledge of the art and science of cooking—for instance—garden vegetables, instead of merely torturing them by fire, needs to be given to boys as well as girls, since until men as well as women have better ideas on the subject, it is not likely that the race will be effectively nourished under our present conditions of civilisation. M. Kriens, the food expert, chef and trainer of boy cooks at the Westminster Technical Institute in Vincent Square, is, by permission of the L.C.C., instructing classes of thirty private cooks in this and other important knowledge. The difference shown by M. Kriens between a cabbage out of which all the healthful salts have been boiled—and thrown away—in which the colour is kept by the unwholesome mixture of soda and the mere fibre sent to table, and the same vegetable cooked in a touch of fat and its own steam is matter for marvel. When, in addition, 2 oz. rice and a gill of water have been added to the pot and cooked for twenty minutes, and, if available, an egg has been poached in a depression made in the surface of the vegetable, it is found that a complete and excellent meal proceeds from one pot. Teachers trained by this expert are now training others to carry on the lessons wherever welcomed. Applications for their help anywhere in the country should be made to the Local Education Authority.

#### WHERE WASTE CAN BE AVOIDED.

But there are other sorts of waste which need checking. Over-use of water, of light, of matches, even of unconsidered trifles such as paper and string, in addition to all things bought for use, whether home-made or imported, wastes some labour, and the "good for trade" excuse has no meaning in the face of life-and-death issues. In fact, it is necessary that the best-sounding excuses should be brushed away. Flowers need not be grown "because some will be sent to hospitals." Grapes or eggs, or the rabbits which are being kept with success even in London, and well fed on food costing 4d. a pound, will be more acceptable now to the wounded. If every elementary school in the kingdom had its rabbit-hutches, bees, and vegetables, how much would be gained!

Thus the point for which education is needed is the translation of the great purpose into terms of daily life. It is "up to" each individual to gain that education and to pass it on. The Ministry of Food will send, on application to Grosvenor House, accredited men and women speakers, who will address meetings, and will especially welcome discussion and suggestion. Such meetings will make available for common use the ideas, suggestions and experiments brought to them by individuals. Incidentally all such propaganda may bring home to the unthinking the question, "Is England worth *my* fighting for?" aud produce an answer in action.

